# Role of frailty in prediction of hospitalized older adult patient’s outcomes: a prospective study

**DOI:** 10.3906/sag-2012-332

**Published:** 2021-10-21

**Authors:** Maryam CHEHREHGOSHA, Reza FADAYE VATAN, Mahtab ALIZADEH-KHOEI, Farshad SHARIFI, Reyhaneh AMINALROAYA, Zahra VAHABI, Abolfazl ZENDEDEL, Hamidreza HEKMAT

**Affiliations:** 1 Department of Gerontology, Faculty of Gerontology, University of Social Welfare and Rehabilitation Sciences, Tehran Iran; 2 Department of Surgical Technology, Faculty of Paramedical Sciences, Golestan University of Medical Sciences, Gorgan Iran; 3 Department of Clinical Gerontology & Geriatric, Faculty of Medicine, Tehran University of Medical Sciences, Tehran Iran; 4 Department of Elderly Health Research, Faculty of Endocrinology and Metabolism Population Sciences, Tehran University of Medical Sciences, Tehran Iran; 5 Department of Geriatric Ziaeian Hospital, Faculty of Medicine, Tehran University of Medical Sciences, Tehran Iran; 6 Department of Memory and Behavioral Neurology Roozbeh Hospital, Faculty of Medicine, Tehran University of Medical Sciences, Tehran Iran; 7 Department of Cardiology Ziaeian Hospital, Faculty of Medicine, Tehran University of Medical Sciences, Tehran Iran

**Keywords:** Frailty, hospitalization, quality of life

## Abstract

**Background/aim:**

Frailty is associated with an increased risk of negative short-term and long-term hospital outcomes. This study aimed to evaluate the role of frailty in predicting readmission, length of stay, and quality of life in the hospitalized older adults.

**Materials and methods:**

This observational study was conducted at Ziaiyan Hospital, Tehran, Iran. In total, 304 participants (65–85 years), were enrolled through the inclusion criteria from August to December 2019. The frailty index (FI) was assessed by the minimum data set-home care. Readmission was obtained through telephone interviews. The length of stay was gathered by the patient’s hospital records, and the EuroQol questionnaire was used for assessing the quality of life. Data were collected by a researcher nurse at the admission time, 30, 60, and 90 days after discharge. The logistic regression model and repeated measures ANOVA were employed to analyze the association between frailty and outcomes.

**Results:**

According to FI, 102 (33.55%) participants were pre-frail, whereas 35 (11.51%) were frail. In the fully-adjusted model for readmission, the pre-frail participants had a higher risk of readmission at the hospital in comparison with the nonfrail and frail groups (OR = 1.88, 95% CI = 1.90–3.26), and also for GP visits, frail patients showed nearly signiﬁcant differences (OR = 2.45, 95% CI = 0.99–6.06) but there were no differences between frail and pre-frail patients in readmissions in the emergency ward. In a fully-adjusted prolonged stay model, pre-frail patients had a higher probability to stay longer in hospital (OR = 2.28, 95% CI: 1.24–4.18). The fully-adjusted model for QoL showed, frail patients were more prone to the declined levels of QoL in comparison with pre-frail patients (OR = 10.77, 95% CI: 3.97–29.18).

**Conclusions:**

The findings indicated that frailty worsened negative outcomes and declined QoL. Early diagnosis in hospital settings could be beneficial for designing optimal care plans for the frail and pre-frail patients.

## 1. Introduction

With the aging population worldwide, the frailty of older adults is a concern for health systems because older patients, especially the frail older adults needing further care and services, are more likely to be hospitalized [1]. Frailty, in the hospitalized older adults, is associated with an increased risk of negative outcomes in the short term (increased length of stay and readmission) and the long term (disability and death) [2]. It could also predict loss of independence, disability, falls, delirium, re-hospitalization, and declined quality of life among the elderly [3]. Although frailty is a common problem in the hospitalized older adult patients, its diagnosis usually faces a few challenges [2]. Currently, there is no consensus for frailty assessment in clinical settings [4].

Clinicians pay close attention to the impact of frailty on health aspects of life among the older adult patients. The right assessment should be applied in proper settings to diagnose frailty accurately [5]. Frailty assessment is performed based on two approaches, i.e. the phenotype model and the cumulative deficit approach. The phenotype approach measures weight loss, fatigue, exhaustion, weakness, physical activity, and mobility dysfunction [6]. Evaluation through the phenotype model can be useful for measuring the functions of the senior citizens such as gait speed and grip strength. However, it is difficult to apply this approach to the older adult patients in hospital settings that may not accurately show the baseline frailty status [4]. 

The other approach is the accumulation of health deficits, known as the frailty index (FI), including 30 or more deficits from different domains related to health [7]. The minimum data set (MDS) assessment form and the comprehensive geriatric assessment (CGA) allow for the measurement of the MDS-specific frailty index and CGA-FI [8]. It is hard to implement the cumulative deficit approach due to a large number of variables; however, some studies indicate that the FI is a more sensitive predictor of adverse outcomes in the older adults because of its multidimensionality [9]. In addition, the FI is a strong predictor of hospital outcomes, mortality, and disability [10]. Hence, the FI might serve as a useful approach to ascertain the effectiveness of health status in clinical settings [11].

Since the MDS-HC form is used as a standard assessment instrument in hospital settings to discharge older patients, the MDS-specific frailty index can be extracted from it [12]. Therefore, the MDS-HC form can be employed to evaluate frailty and detect short-term and long-term outcomes among the hospitalized older adult patients [4]. This study aims to appraise the role of frailty in the prediction of patients outcomes (readmission, length of stay, and quality of life) among the hospitalized older adults based on the MDS-specific frailty index.

## 2. Material and methods 

### 2.1. Study design and participants

This prospective observational study was conducted on geriatric patients (n = 304) who were admitted to Ziaiyan Hospital (an educational hospital affiliated with Tehran University of Medical Sciences) from August 2019 to December 2019.

Since the minimum data set-home care (MDS-HC) requires accurate responses of the older adults and their caregivers, they were selected through the following criteria 1) The patients were aged between 65 and 85 years old. 2) They were admitted to geriatric, internal, and coronary care unit wards. 3). They were not admitted to ICU. 4). They were not terminal ill or in high need of care. 5). They did not reside in a nursing home. 6). The presence of a caregiver was mandatory for the consent of patients with a lack of mental capacity. 

Severe disease cases or the older adult who were transferred from hospitals to nursing homes were excluded, for they were unable to fill out the questionnaires or complete functional assessments. The health-related and functional variables were collected through face-to-face interviews conducted by a trained nurse at admission time based on the MDS-HC.

Informed consent was obtained from patients or their legally acceptable representatives. The study was approved by the Ethics Committee of the University of Social Welfare and Rehabilitation (IR.USWR.REC.1396.296). The frailty index and other outcomes base on the following information were extracted.

### 2.2. Frailty assessment

The MDS-HC is a standard geriatric assessment tool that contains more than 200 items regarding attention, cognition, orientation, mood and affection, function, nutrition, medication, pain, incontinence, and environment. In this study, the frailty index (FI) was constructed by using 42 health-related deficits/variables based on an FI derived from the MDS-HC. More information about the calculation of the FI was written in Burn et al. study [13]. To calculate the FI, it was necessary to answer all 42 health-related deficits/variables, so the incomplete information of the older adults was not considered in the calculation of the FI, and they were excluded from the study. Each variable was recorded on a binary scale of 0 or 1 (1 represents the presence of a deficit, whereas 0 represents the absence of a deficit). The FI was calculated by adding up the number of deficits recorded for a patient. The summation was then divided by the total number of possible deficits representing an FI with a potential range from 0 to 1 [13]. In this analysis, like the study by McKenzie et al., three frailty categories were obtained: nonfrail (≤0.21), pre-frail (>0.21 to ≤0.30), and frail (>0.30) [14].

### 2.3. Readmission information

Readmission is defined as at least another admission to a hospital or an emergency ward or a visit to a general practitioner (GP) for any reason within 3 months after discharge [15]. Readmission information was obtained from all patients through telephone interviews conducted by a trained nurse within 30, 60, and 90 days from the baseline.

### 2.4. Length of stay information

The length of stay was defined as the number of days between admission and discharge (or death). The prolonged hospitalization period was also calculated for further analysis based on the following definition: “A prolonged length of stay is equal to or greater than 75% of the total length of stay in the entire cohort study” [16].

### 2.5. Quality of life assessment

The EuroQol five-dimensional questionnaire (EQ-5D-3L) was used for assessing the quality of life. This tool consists of two parts, i.e. the EQ-5D descriptive system and the EQ-5D visual analog scale (EQ-5D VAS). The EQ-5D descriptive system includes mobility, self-care, usual activities, pain/discomfort, and anxiety/depression. The total score ranges from –0.594 to 1 based on the UK weighted index [17]. Validation of an Iranian version of “EQ-5D-3L” questionnaire has been done by Dastourani et al. study [18].

In consistency with the study of Parkin et al., the results were classified as two categories in this study to define the QoL score as low quality of life (≤0.50) and high quality of life (>0.50) [19]. In the EQ-5D VAS, respondents registered the self-rated health on a vertical visual analog scale, ranging from 0 (the worst health status) to 100 (the best health condition) [17,19].

### 2.6. Co-variables

The information of age, sex, educational attainment, marital status, co-morbidity, polypharmacy, cognition, and depression was collected to evaluate the impacts of variables that were not encoded directly in the FI. Co-morbidity is defined as the co-existence of at least 3 separate chronic illnesses [20]. 

Poly-pharmacy is defined as the concurrent use of more than 5 medications [21]. The patients’ cognitive states were evaluated by conducting the six-item cognitive impairment test (6-CIT) consisting of orientation, attention, and memory domains. The score ranges from 0 to 28, and scores higher than 11 indicate cognitive impairment [22].

Depression was measured by the MDS-depression rating scale (MDS-DRS) with a maximum score of 14. The patient’s MDS-DRS score was interpreted based on the following category, i.e. nondepression (0), mild/moderate depression (<1 to >3), and severe depression (<3) [23]. A binary classification was used for the logistic regression. It included two categories (depression≥3 and without depression <3) [24]. 

### 2.7. Statistical analyses

Statistical analysis was performed in SPSS v.16.0 (SPSS Inc., Chicago, IL, USA) and Stata 11 (Stata Corp., College Station, TX) at p-values <0.05 (two-tailed). The normal distribution of continuous variables was assessed by conducting the Kolmogorov–Smirnov test. The continuous and categorical variables were presented as a mean (± standard deviations) and numbers or proportions, respectively. The discrimination of frail, pre-frail, and nonfrail groups was tested through analysis of variance (ANOVA). The categorical variables were compared by using Chi-squared tests. Furthermore, the unadjusted and fully-adjusted logistic regression models were employed to estimate the odds ratio (OR) and its 95% confidence interval (CI) in frailty status as an independent variable. A repeated measure ANOVA was conducted to check the trends in quality of life over time. 

## 3. Results

A total of 304 geriatric patients agreed to participate in this study; however, 16 participants were excluded due to incomplete assessment resulting from follow-up inability or death. 

The mean age of the older adult patients was about 75.72 ± 6.30 years. The FI maximum score was reported at 0.540, and the mean ± SD of FI scores was reported 0.21 ± 0.08. Based on FI cutoff points, 102 (33.55%) patients were identified as pre-frail, whereas 35 (11.51%) were diagnosed as frail. 

The frail patients were older than the pre-frail and nonfrail (78.2 ± 6.41 vs. 76.43 ± 5.93 and 74.76 ± 6.32; p = 0.004) patients. There were significant differences between variables (age, co-morbidity, depression, cognition, quality of life, readmission, and prolonged stay) in frailty status [Table 1].

**Table 1:** Sociodemographic and disease characteristics of older adult patients according to the frailty level (n = 304).

Variables		Nonfrail (N = 167)	Pre-frail (N = 102)	Frail(N = 35)	P-value
Age		74.76 ± 6.32	76.43 ± 5.93	78.2 ± 6.41	0.004
Sex					0.678
Male		44 (26.35)	28 (27.45)	7 (20.00)	
Female		123 (73.65)	74 (72.55)	28 (80.00)	
Education level					0.275
Illiterate		0 (0)	1 (0.98)	1 (2.86)	
Primary school		102 (61.08)	71 (69.61)	28 (80.00)	
Secondary school or advanced		65 (38.92)	30 (29.41)	6 (17.14)	
Marital status					0.656
Single		2 (1.20)	0 (0)	0 (0)	
Married		77 (46.11)	48 (47.06)	12 (34.29)	
Widow/divorce		88 (52.70)	54 (52.94)	23 (65.71)	
Polypharmacy [20]					0.130
5< Drug		27 (16.17)	10 (9.80)	2 (5.71)	
5> Drug		140 (83.83)	92 (90.20)	33 (94.29)	
Co-morbidity [19]					0.021
3< DiseaseZ-score value		93 (55.69)2.57	39 (38.24)–2.67	17 (48.57)–0.05	
3> DiseaseZ-score value		74 (44.31)–2.57	63 (61.76)2.67	18 (51.43)0.05	
Depression (MDS- DRS1-)					< 0.001
NormalZ-score value		88 (52.69)5.16	24 (23.53)–4.07	8 (23.53)–2.03	
Mild/moderateZ-score value		57 (34.13)1.15	32 (31.37)0.00	6 (17.65)–1.82	
SevereZ-score value		22 (13.17)–6.74	46 (45.10)4.38	20 (58.82)4.05	
Cognition (6 CIT)2		5.05 ± 3.81	6.78 ± 3.83	9.62 ± 3.88	< 0.001
Frailty index		0.16 ± .03	0.25 ± 0.02	0.38 ± 0.06	-
Length of stay		6.03 ± 2.65	7.62 ± 3.91	7.74 ± 3.72	0.064
Prolonged stay [16]					0.001
> 8daysZ-score value		137 (82.04)3.77	63 (61.76)–3.24	23 (65.71)–1.08	
<8 daysZ-score value		30 (17.96)–3.77	39 (38.24)3.24	12 (34.29)1.08	
Quality of life					< 0.001
EQ5D3		0.68 ± .25	0.49 ± .31	0.26 ± 0.34	
EQ.VAS4		55.14 ± 15.68	44.21 ± 15.86	38.42 ± 12.58	< 0.001
Readmission					
HospitalZ-score value	No	23 (14.37)2.61	2 (2.17)–3.10	4 (12.90)0.51	0.008
	Yes	137 (85.63)–2.61	90 (97.83)3.10	27 (87.10)–0.051	
Emergency departmentZ-score value	No	101 (60.48)1.51	48 (47.06)–2.37	23 (65.71)1.15	0.050
	Yes	66 (39.52)–1.51	54 (52.94)2.37	12 (34.29)–1.15	
GPs. visitZ-score value	No	100 (59.88)–0.25	75 (73.53)3.29	9 (25.71)–0.4.47	< 0.001
	Yes	67 (40.12)0.25	27 (26.47)–3.29	26 (74.29)4.47	

### 3.1. The relationship between frailty status and readmission

Unadjusted logistic regression analysis showed significant differences between nonfrail and pre-frail patients in readmission rates at the hospital (unadjusted OR = 2.12, 95% CI = 1.27–3.54) and emergency ward (unadjusted OR = 1.72, 95% CI = 1.04–2.83). Based on results regarding frail patients compared with nonfrail and pre-frail patients, the GP visits were highly significant (unadjusted OR = 4.31, 95% CI = 1.90–9.77).

After age, sex, depression, and cognition variables were adjusted, the pre-frail participants had a higher risk of readmission at the hospital in comparison with the nonfrail and frail groups (fully-adjusted OR = 1.88, 95% CI = 1.90–3.26). In the fully-adjusted model for the emergency ward variables, there were no significant differences between frail and pre-frail patients in readmissions. In the fully-adjusted model for GP visits, frail patients showed nearly signiﬁcant differences (fully-adjusted OR = 2.45, 95% CI = 0.99–6.06). In fully-adjusted logistic regression, sex (male) and cognitive impairment variables increased the emergency ward readmissions frequency of the elderly patients. Moreover, the pre-frail and depressed patients were more prone to GP visits in this study (Table 2).

**Table 2:** Unadjusted and full adjusted logistic regression analysis of frailty status and related factors.

Variables		Odds	CI (95%)	P-value
Prolonged stay	Unadjusted			
	Nonfrail	Reference		
	Pre-frail	2.826	1.611–4.957	< 0.001
	Frail	2.382	1.068–5.313	0.034
	Fully adjusted			
	Nonfrail	Reference		
	Pre-frail	2.280	1.241–4.185	0.008
	Frail	1.457	0.584–3.633	0.419
	Age	1.023	0.977–1.070	0.320
	Sex (Male/Female)	2.084	1.068–4.067	0.031
	Depression (Nondepressed/depressed)	1.755	0.958–3.214	0.068
	Cognition (Not impaired/impaired)	1.274	0.718–2.261	0.408
Readmission (GPs_vist)	Unadjusted			
	Nonfrail	Reference		
	Pre- frail	.537	0.313–0.920	0.024
	Frail	4.311	1.901–9.777	< 0.001
	Fully adjusted			
	Nonfrail	Reference		
	Pre- frail	9.359	0.194–0.666	0.001
	Frail	2.458	0.996–6.068	0.051
	Age	1.002	0.962–1.044	0.894
	Sex (Male/Female)	1.097	0.625–1.924	0.745
	Depression (Nondepressed/depressed)	2.540	1.387–4.650	0.003
	Cognition (Not impaired/impaired)	1.404	0.822–2.396	0.213
Readmission (Emergency department)	Unadjusted			
	Nonfrail	Reference		
	Pre- frail	1.721	1.046–2.831	0.032
	Frail	0.798	0.371–1.713	0.563
	Fully adjusted			
	Nonfrail	Reference		
	Pre-frail	1.618	0.939–2.787	0.083
	Frail	0.652	0.277–1.534	0.328
	Age	0.992	0.954–1.032	0.719
	Sex (Male/Female)	0.568	0.335–0.963	0.036
	Depression (Nondepressed/depressed)	1.014	0.577–1.781	0.961
	Cognition (Not impaired/impaired)	1.670	1.005–2.776	0.048
Readmission (hospital)	Unadjusted			
	Nonfrail	Reference		
	Pre-frail	2.123	1.273–3.541	0.004
	Frail	1.260	0.606–2.618	0.534
	Fully adjusted			
	Nonfrail	Reference		
	Pre-frail	1.887	1.090–3.267	0.023
	Frail	0.964	0.423–2.196	0.932
	Age	0.998	0.960–1.038	0.945
	Sex (Male/Female)	1.044	0.616–1.770	0.870
	Depression (Nondepressed/depressed)	1.289	0.731–2.271	0.379
	Cognition (Not impaired/impaired)	1.354	0.815–2.250	0.241
Quality of life (EQ5D)	Unadjusted			
	Nonfrail	Reference		
	Pre-frail	5.718	3.177–10.29	< 0.001
	Frail	16.44	6.63–40.70	< 0.001
	Fully adjusted			
	Nonfrail	Reference		
	Pre-frail	4.941	2.630–9.280	< 0.001
	Frail	10.77	3.976–29.183	< 0.001
	Age	1.054	1.004–1.106	0.031
	Sex (Male/Female)	1.408	0.736–2.696	0.301
	Depression (Nondepressed/depressed)	1.241	0.643–2.393	0.519
	Cognition (Not impaired/impaired)	1.684	0.935–3.032	0.082

### 3.2. The Relationship between frailty status and length of stay 

There were no significant differences between nonfrail, pre-frail, and frail geriatric patients in the length of stay. In the unadjusted logistic regression model, pre-frail (unadjusted OR = 2.82, 95% CI = 1.61–4.95) and frail patients (unadjusted OR = 2.38, 95% CI =1.06–5.31) were more prone to prolonged stay at the hospital. In a fully-adjusted prolonged stay model, pre-frail geriatric patients had a higher probability to stay longer in hospital (fully-adjusted OR =2.28, 95% CI: 1.24–4.18); however, pre-frail and frail elderly women experienced higher levels of the length of stay in hospital [Table 2].

### 3.3. The relationship between frailty status and quality of life 

The unadjusted logistic regression model showed significant differences between nonfrail, frail (unadjusted OR = 16.44, 95% CI = 6.63–40.70), and pre-frail elderly patients in the scores of QoL (unadjusted OR = 5.71, 95% CI = 3.17–10.29). In the fully-adjusted model, frail patients (fully-adjusted OR = 10.77, 95% CI: 3.97–29.18) were more prone to the declined levels of QoL in comparison with pre-frail patients. Furthermore, QoL can decline more in older frail or pre-frail patients (Table 2).

The results of repeated measures ANOVA showed significant differences in QoL scores among nonfrail, pre-frail, and frail older adult patients at the baseline 30, 60, and 90 days after discharge from the hospital (p < 0.001) (Table 3). 

**Table 3:** Comparison of the average scores of EQ5D and EQ. VAS at the baseline and three times assessments, based on repeated measures ANOVA.

Variable		Baseline	30 days	60 days	90 days	P-value1	P-value2
EQ5D	Nonfrail	0.68 ± 0.24	0.66 ± 0.24	0.65 ± 0.24	0.65 ± 0.24	< 0.001	< 0.001
	Pre frail	0.49 ± 0.31	0.42 ± 0.31	0.40 ± 0.30	0.38 ± 0.31		
	Frail	0.27 ± 0.35	0.17 ± 0.32	0.13 ± 0.31	0.12 ± 0.31		
EQ.VAS	Nonfrail	55.19 ± 15.70	54.54 ± 15.44	54.17 ± 15.45	54.10 ± 15.48	< 0.001	< 0.001
	Pre frail	44.20 ± 15.86	40.80 ± 15.21	39.95 ± 14.92	39.73 ± 14.87		
	Frail	38.28 ± 12.92	33.59 ± 12.39	32.19 ± 11.70	31.09 ± 11.69		

Figure shows the descending slope of QoL (EQ5D and EQ.VAS) scores at baseline, 30, 60, and 90 days after geriatric patients were discharged from the hospital.

**Figure:**
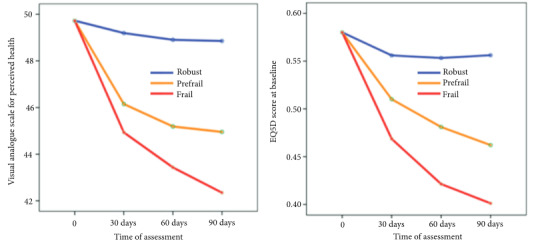
Trend of EQ5D and EQ.VAS score at the base line and three-time assessment.

## 4. Discussion

This study described that the significant differences in specific variables (age, co-morbidity, depression, cognition, quality of life, readmission, and prolonged stay) concerning the frailty status of geriatric inpatients. Furthermore, a significant association was observed between frailty and prolonged stay, readmission, and QoL among the hospitalized older adult patients. The prolonged stay was prominent in pre-frail geriatric patients in the hospital. In frail geriatric patients, the probability of a GP visit was approximately significant. The QoL was declined in frail, pre-frail, and nonfrail patients during the 3-month follow-up; it decreased more in frail patients. 

Based on results in the present study, there were more readmissions in frail and pre-frail geriatric patients. Similarly, Vidan et al. reported that frailty was an independent predictor in the hospitalized Spanish older adults within 12 months of readmission [25]. 

In hospitalized Chinese patients, frailty increased the risk of readmission [26]. Additionally, in aortic valve implant older adult patients in a Japanese study, frailty was correlated with unplanned readmission [27]. The major reason for an association between frailty and readmission might be the assumption that patients admitted to hospitals were more sensitive to frailty syndrome and experienced a higher risk of readmission or poor outcomes [28]. At the same time, the discharge process might not consider the health-related concerns and needs of the older adult patients in some hospitals [29]; therefore, it may increase the chance of re-hospitalization among frail and pre-frail older patients.

Results showed that cognitive impairment and sex [30] affected readmissions in frail patients. The present study also indicated higher emergency ward readmissions in men as well as cognitive impairment in frail and pre-frail patients. Existing sex differences in the findings might be attributed to health-seeking behavior and perceived health status. The majority of the older adult men were less interested in using follow-up care and preventive programs. They were also more prone to unintentional acute illnesses because of unwillingness to comply with preventive programs. This could explain the more ED readmissions in older males after discharge [31]. 

Possible mechanisms for increasing the probability of re-admission in cognitive impairment patients might be due to disorientation in the time or place as well as problems in complying with simple commands in the hospital as a result of attention/memory deficits [32]. Moreover, patients and caregivers are usually agitated concerning the issues that will emerge after discharge. Patients commonly fail to pay attention to the necessary instructions after discharge [33]; therefore, they are re-admitted quickly after discharge.

The findings of this study showed that the probability of prolonged stay was higher in the pre-frail hospitalized older adults. This finding is consistent with the results of other studies [30,34]. Apparently, the Iranian pre-frail older adult patients were more prone to lengthy hospital stay [35]. This might be because the frail patients were mostly bed-ridden in their homes [35] due to being mistreated by their family caregivers or facing ageism taboo, which might have been neglected by their family caregivers [36]. The readmission rate was lower in the elderly frail patients than in the pre-frail patients.

According to the findings, the older adult women were more likely to stay in hospital. This was consistent with the findings reported by De Buyser et al. [37]. However, Alnajashia et al. [38] found no significant association between length of stay and sex. It might be due to higher levels of life expectancy in the older adult women, compared with older men, as well as the high probability to live alone and the high rate of co-morbidity in the Iranian older female than the male older adults [39]. Besides, there is no social security system in Iran to support the elderly (in terms of financial and career services), especially for older women. As a result, the older women may stay longer in hospital.

Based on the research findings, the older adult frail patients had a lower QoL score. In a similar study, Cavrini et al. reported that the QoL score was correlated with the number of hospitalization and institutionalization in the Italian older adults within two years of follow-up [40]. Kahlon et al. observed that frail patients had lower QoL scores than nonfrail older adult patients in Canada [41]. In contrast, Kojima et al. noted that the British pre-frail older adult patients not only had a better QoL score at baseline but also showed improvements in QoL over time [42]. However, the research settings of our study are not similar to those of the reviewed studies.

In this study, the reason for a lower score of QoL in frail older adult patients might be interpreted as the fact that hospitalization reduced the mobility and functional capacity of the older adults and increased dependency [43]. Meanwhile, independence, and self-care are important measures in the lives of the older adults which are disrupted during hospitalization; thus, it appears that hospitalization decreased QoL in frail patients.

There were a few research limitations. This is a single-center study, the findings of which might not be generalizable. Only one frailty assessment tool (MDS-HC frailty index) was employed due to its practicality, ease of administration, and complete assessment of multiple important geriatric domains. The cause and duration of each readmission were not discussed in the study evaluation intervals.

In the present study, the frailty assessment was performed using MDS-HC in the hospital setting, since adopting frailty measures depends greatly on clinical settings and the purpose of frailty assessment [26]. The results obtained revealed that the MDS-specified frailty index was able to predict the adverse outcomes in the hospitalized older adult patients. Based on the MDS-specified frailty index, the pre-frail status was more prevalent among geriatric inpatients. This is a valuable finding for policymakers so that they can be aware of the vulnerable older adult population in Iran and prepare appropriate care plans for major inpatient vulnerable groups. 

## Informed consent

Informed consent was obtained from all patients or their legally acceptable representatives. The study was approved by the Ethics Committee of the University of Social Welfare and Rehabilitation(IR.USWR.REC.1396.296).

## References

[ref1] Kim DH 2020 Measuring frailty in health care databases for clinical care and research Annals Geriatric Medicine Research 24 62 74 10.4235/agmr.20.0002 PMC7370795 32743326

[ref2] Hubbard RE Peel NM Samanta M Gray LC Mitnitski A 2017 Frailty status at admission to hospital predicts multiple adverse outcomes Age Ageing 46 801 806 28531254 10.1093/ageing/afx081

[ref3] ’Hoski O Bean S Ma JF So J Kuspinar HY 2020 Physical function and frailty for predicting adverse outcomes in older primary care patients Archives of Physical Medicine and Rehabilitation 101 592 598 31891711 10.1016/j.apmr.2019.11.013 PMC7103496

[ref4] Chong E Ho E Baldevarona-Llego J Chan M Wu L 2018 Frailty in hospitalized older adults: comparing different frailty measures in predicting short- and long-term patient outcomes Journal of the American Medical Directors Association 19 450 457 29153536 10.1016/j.jamda.2017.10.006

[ref5] Chen CL Chen CM Wang CY Ko PW Chen CH 2019 Frailty is associated with an increased risk of major adverse outcomes in elderly patients following surgical treatment of hip fracture Scientific Reports 9 1 9 31836751 10.1038/s41598-019-55459-2 PMC6910954

[ref6] Lachmann R Stelmach-Mardas M Bergmann MM Bernigau W Weber D 2019 The accumulation of deficits approach to describe frailty PLoS One 14 10.1371/journal.pone.0223449 PMC6793873 31613904

[ref7] Searle SD Mitnitski A Gahbauer EA Gill TM Rockwood K. 2008 A standard procedure for creating a frailty index BMC Geriatrics 8 24 24 18826625 10.1186/1471-2318-8-24 PMC2573877

[ref8] Wellens NIH Deschodt M Flamaing J Moons P Boonen S 2011 First-generation versus third-generation comprehensive geriatric assessment instruments in the acute hospital setting: a comparison of the minimum geriatric screening tools (MGST) and the interRAI acute care (interRAI AC) The Journal of Nutrition, Health and Aging 15 638 44 10.1007/s12603-011-0109-221968858

[ref9] Rockwood K Andrew M 2007 Mitnitski A. A comparison of two approaches to measuring frailty in elderly people The Journals of gerontology. Series A, Biological Sciences and Medical Sciences 62 738 743 10.1093/gerona/62.7.738 17634321

[ref10] Kerminen H. Huhtala H. Jäntti P. 2020 Frailty index and functional level upon admission predict hospital outcomes: an interRAI-based cohort study of older patients in post-acute care hospitals BMC Geriatrics 20 1 12 10.1186/s12877-020-01550-7 PMC7201739 32370740

[ref11] Cesari M Gambassi G Van Kan GA Vellas B. 2014 The frailty phenotype and the frailty index: different instruments for different purposes Age Ageing 43 10 2 24132852 10.1093/ageing/aft160

[ref12] Gray LC Bernabei R Berg K Finne-Soveri H Fries BE 2008 Standardizing assessment of elderly people in acute care: the interRAI acute care instrument Journal of the American Geriatrics Society 56 536 541 18179498 10.1111/j.1532-5415.2007.01590.x

[ref13] Burn R Hubbard RE Scrase RJ Abey-Nesbit RK Peel NM 2018 A frailty index derived from a standardized comprehensive geriatric assessment predicts mortality and aged residential care admission BMC Geriatrics 18 319 319 30587158 10.1186/s12877-018-1016-8 PMC6307300

[ref14] McKenzie K Ouellette-Kuntz H Martin L 2015 Using an accumulation of deficits approach to measure frailty in a population of home care users with intellectual and developmental disabilities: an analytical descriptive study public health, nutrition and epidemiology BMC Geriatrics 15 10.1186/s12877-015-0170-5 PMC4683739 26678519

[ref15] Brennan J Chan T Killeen J Castillo E. 2015 Inpatient readmissions and emergency department visits within 30 days of a hospital admission Western Journal of Emergency Medicine 16 1025 1029 10.5811/westjem.2015.8.26157 PMC4703150 26759647

[ref16] Nguyen TN Cumming RG Hilmer SN 2016 The impact of frailty on mortality, length of stay and re-hospitalisation in older patients with atrial fibrillation Heart, Lung and Circulation 25 551 557 10.1016/j.hlc.2015.12.002 26809464

[ref17] Rabin R De Charro F. EQ 2001 -5D: a measure of health status from the EuroQol group Annals of Medicine 33 337 343 11491192 10.3109/07853890109002087

[ref18] Dastourani A Sohani SM Ali SS Dehkordi SN 2019 Reliability and validity of the persian version of the european quality of life questionnaire (EQ-5D-3L) in patients with meniscus and knee ligaments injury The Journal of Paramedical Science and Rehabilitation 7 73 82

[ref19] Parkin D Devlin N Feng Y. 2016 What determines the shape of an EQ-5D index distribution? Medical Decision Making 36 941 951 10.1177/0272989X16645581 27112934

[ref20] Caughey GE Vitry AI Gilbert AL Roughead EE 2008 Prevalence of comorbidity of chronic diseases in Australia BMC Public Health 8 221 221 18582390 10.1186/1471-2458-8-221 PMC2474682

[ref21] Morin L Johnell K Laroche M-L Fastbom J Wastesson JW 2018 The epidemiology of polypharmacy in older adults: register-based prospective cohort study Clinical Epidemiology 10 289 298 29559811 10.2147/CLEP.S153458 PMC5856059

[ref22] Katzman R Brown T Fuld P Peck A Schechter R 1983 Validation of a short orientation-memory-concentration test of cognitive impairment American Journal of Psychiatry 140 734 739 10.1176/ajp.140.6.734 6846631

[ref23] Burrows AB Morris J Simon S Hirdes J Phillips C 2000 Development of a minimum data set-based depression rating scale for use in nursing homes Age Ageing 29 165 172 10791452 10.1093/ageing/29.2.165

[ref24] Hogeveen SE Chen J Hirdes JP 2017 Evaluation of data quality of interRAI assessments in home and community care BMC Medical Informatics and Decision Making 17 150 150 29084534 10.1186/s12911-017-0547-9 PMC5663080

[ref25] Vidán MT Blaya-Novakova V Sánchez E Ortiz J Serra-Rexach JA 2016 Prevalence and prognostic impact of frailty and its components in non-dependent elderly patients with heart failure European Journal of Heart Failure 18 869 875 27072307 10.1002/ejhf.518

[ref26] Hao Q Zhou L Dong B Yang M Dong B 2019 The role of frailty in predicting mortality and readmission in older adults in acute care wards: a prospective study Scientific Reports 9 1207 1207 30718784 10.1038/s41598-018-38072-7 PMC6362215

[ref27] Saji M Higuchi R Tobaru T Iguchi N Takanashi S 2018 Impact of frailty markers for unplanned hospital readmission following transcatheter aortic valve implantation Circulation Journal 82 2191 2198 29311518 10.1253/circj.CJ-17-0816

[ref28] Dharmarajan K Hsieh AF Lin Z Bueno H Ross JS 2013 Diagnoses and timing of 30-day readmissions after hospitalization for heart failure, acute myocardial infarction, or pneumonia The Journal of the American Medical Association 309 355 363 23340637 10.1001/jama.2012.216476 PMC3688083

[ref29] Caleres G Bondesson Å Midlöv P Modig S. 2018 Elderly at risk in care transitions when discharge summaries are poorly transferred and used -a descriptive study BMC Health Services Research 18 770 770 30305104 10.1186/s12913-018-3581-0 PMC6180642

[ref30] Bernabeu-Mora R García-Guillamón G Valera-Novella E Giménez-Giménez LM Escolar-Reina P 2017 Frailty is a predictive factor of readmission within 90 days of hospitalization for acute exacerbations of chronic obstructive pulmonary disease: a longitudinal study Therapeutic Advances in Respiratory Disease 11 383 392 28849736 10.1177/1753465817726314 PMC5933665

[ref31] Kirchengast S Haslinger B 2008 Gender differences in health-related quality of life among healthy aged and old-aged Austrians: cross-sectional analysis Gender Medicine 5 270 278 18727993 10.1016/j.genm.2008.07.001

[ref32] Goldberg SE Harwood RH 2013 Experience of general hospital care in older patients with cognitive impairment: are we measuring the most vulnerable patients’ experience? BMJ Quality & Safety 22 977 980 10.1136/bmjqs-2013-001961 PMC3888593 23868868

[ref33] Coleman EA Berenson RA 2004 Lost in transition: challenges and opportunities for improving the quality of transitional care Annals of Internal Medicine 141 533 536 15466770 10.7326/0003-4819-141-7-200410050-00009

[ref34] Hope J Recio-Saucedo A Fogg C Griffiths P Smith GB 2018 A fundamental conflict of care: nurses’ accounts of balancing patients’ sleep with taking vital sign observations at night Journal of Clinical Nursing 27 1860 1871 29266489 10.1111/jocn.14234 PMC6001445

[ref35] Juma S Taabazuing M-M Montero-Odasso M. 2016 Clinical frailty scale in an acute medicine unit: a simple tool that predicts length of stay Canadian Geriatrics Journal 19 34 39 27403211 10.5770/cgj.19.196 PMC4922366

[ref36] 2015 Reports of elder neglect by older adults, their family caregivers, and their home care workers: a test of measurement invariance The Journals of Gerontology Series B: Psychological Sciences and Social Sciences 70 432 442 24859223 10.1093/geronb/gbu051

[ref37] De Buyser SL Petrovic M Taes YE Vetrano DL Onder G. 2014 A multicomponent approach to identify predictors of hospital outcomes in older in-patients: a multicentre, observational study PLoS One 26 10.1371/journal.pone.0115413 PMC4277310 25542042

[ref38] Alnajashia MA Almasouda MA Aldaham SA Acuna JM Zevallos JC 2016 Association of gender and length of stay among Puerto Ricans hospitalized with decompensated heart failure Medicine (United States) 95 10.1097/MD.0000000000004255 PMC5265772 27442655

[ref39] Alimohammadian M Majidi A Yaseri M Ahmadi B Islami F 2017 Multimorbidity as an important issue among women: results of a gender difference investigation in a large population-based cross-sectional study in West Asia BMJ Open 10 10.1136/bmjopen-2016-013548 PMC5623450 28490550

[ref40] Cavrini G Broccoli S Puccini A Zoli M. 2012 EQ-5D as a predictor of mortality and hospitalization in elderly people Quality Life Research 21 269 280 10.1007/s11136-011-9937-0 21656336

[ref41] Kahlon S Pederson J Majumdar SR Belga S Lau D 2015 Association between frailty and 30-day outcomes after The Canadian Medical Association Journal 187 799 804 26009583 10.1503/cmaj.150100 PMC4527901

[ref42] Kojima G Iliffe S Morris RW Taniguchi Y Kendrick D 2016 Frailty predicts trajectories of quality of life over time among British community-dwelling older people Quality Life Research 25 1743 1750 10.1007/s11136-015-1213-2 PMC4893360 26747318

[ref43] Carvalho TC Valle AP Jacinto AF Mayoral VF Boas PJFV 2018 Impact of hospitalization on the functional capacity of the elderly: a cohort study Revista Brasiliera de Geriatria e Gerontologia 21 134 142

